# Understanding the Link Between Physical Activity and Work Ability in University Staff: Protocol for a Gender-Sensitive Cross-Sectional Study

**DOI:** 10.2196/80298

**Published:** 2025-08-28

**Authors:** Laura Lorenzo-Gallego, Silvia Muñoz-Pastor, Maria Remedios Menéndez-Calvo, Beatriz Navarro-Brazález, María Torres-Lacomba

**Affiliations:** 1 Physiotherapy in Women’s Health Research Group Nursing and Physiotherapy Department Universidad de Alcalá Alcalá de Henares (Madrid) Spain; 2 Ramón y Cajal Institute of Health Research Hospital Universitario Ramón y Cajal Madrid Spain; 3 Research Group on Gender Equality Legal Sciences Department Universidad de Alcalá Alcalá de Henares (Madrid) Spain

**Keywords:** physical activity, sedentary behavior, working conditions, occupational health, gender perspective, leisure activities

## Abstract

**Background:**

Physical inactivity represents a significant public health issue with substantial socioeconomic costs. In the autonomous Community of Madrid, 39.17% of the population does not meet the World Health Organization recommendations for physical activity (PA). Gender, sex, and occupational factors are well-established determinants of leisure-time physical activity (LTPA); yet, few studies have examined these factors among university staff.

**Objective:**

This study aims to analyze the relationship between LTPA and work ability among university staff in the autonomous Community of Madrid, considering the potential modifying effect of occupational PA. Secondary objectives include examining associations between LTPA, musculoskeletal disorders, health-related quality of life, physical and mental workload, and working conditions, with a focus on sex and gender differences.

**Methods:**

A cross-sectional study was designed involving 885 university staff members from the University of Alcalá, Madrid, Spain. Participants will complete an online survey, including sociodemographic questions and validated instruments: the Global Physical Activity Questionnaire, Work Ability Index, Nordic Musculoskeletal Questionnaire, Short Form-12 Health Survey, and the National Aeronautics and Space Administration Task Load Index. Descriptive and inferential statistics will be performed to assess the associations between LTPA, occupational PA, and work ability, adjusted for relevant covariates.

**Results:**

This study was approved by the ethics committee of the University of Alcalá in November 2024. Recruitment began in December 2024 and will continue until June 2027. Data analysis will be conducted progressively. Results will be disseminated in peer-reviewed journals and presented at scientific conferences following gender-sensitive and transparent reporting standards.

**Conclusions:**

Understanding the determinants of PA and their interaction with work ability and gender may inform the development of targeted, culturally sensitive interventions to reduce sedentary behavior and its associated health and economic burdens in university staff.

**Trial Registration:**

ClinicalTrials.gov NCT06723808; https://clinicaltrials.gov/study/NCT06723808

**International Registered Report Identifier (IRRID):**

DERR1-10.2196/80298

## Introduction

### Background

Physical activity (PA) is widely recognized as a cornerstone of public health due to its proven effectiveness in preventing and managing noncommunicable diseases [1]. However, PA has emerged as an escalating global health and socioeconomic challenge [2], significantly impacting the Spanish population, particularly in the autonomous Community of Madrid (CAM), where more than 39% of the adults fail to meet World Health Organization (WHO) guidelines [3]. In occupational contexts, this is especially concerning due to the negative association between inactivity and work ability, a key predictor of productivity, employment sustainability, and healthy aging in the workforce [4,5].

PA can be classified into different domains, with leisure-time physical activity (LTPA) and occupational physical activity (OPA) being particularly relevant in work-related health research [6,7]. OPA refers to the physical effort involved in work-related tasks [8], whereas LTPA encompasses voluntary activities, such as exercise, sports, and recreation [6]. Although both contribute to total PA, their effects on health are not equivalent [4,6-9].

Substantial evidence links high levels of LTPA with enhanced overall health, lower risk of noncommunicable diseases, and improved mental and musculoskeletal health [1,9]. Conversely, high OPA levels have been associated with increased risks of musculoskeletal disorders (MSDs) [10], cardiovascular diseases [10,11], and higher mortality rates. This apparent contradiction, referred to as the “PA paradox,” highlights that OPA often lacks sufficient intensity, involves prolonged durations without recovery, and typically occurs under low-autonomy conditions [11]. This paradox provides a relevant conceptual framework for this study, reinforcing the importance of examining LTPA and OPA as distinct domains with potentially divergent effects on work ability.

Moreover, while LTPA has been shown to positively influence occupational outcomes, including work ability [12], reduced absenteeism [13,14], greater job satisfaction [15], improved performance, and higher productivity [16,17], an imbalance between OPA and LTPA may lead to diminished functional capacity, increased absenteeism, and an elevated risk of burnout [11-13,17-21].

Although international guidelines, such as those issued by the WHO, define minimum PA thresholds for various age groups and conditions [20], social determinants of health, including gender, sex, socioeconomic status, and workplace environment, play a significant role in shaping individuals’ ability to meet these recommendations [22-29]. In this context, unequal access to LTPA opportunities contributes to the persistence of health disparities [20,21].

Gender and sex play a particularly important role in shaping PA behavior and work-related outcomes. Women tend to engage in less LTPA than men, a pattern observed in Spain and the CAM region, where 46.89% of the women report being sedentary compared to 30.61% of the men [3]. This disparity is likely driven by a combination of gender- and sex-related factors. Gender-based determinants may include unequal caregiving responsibilities, reduced time for leisure due to social roles, and cultural expectations regarding self-care and productivity, which can limit women’s participation in LTPA [22,23,30]. In contrast, sex-related factors, such as menopause, endometriosis, and urinary incontinence (biological or physiological processes specific to female reproductive health), may directly impact physical capacity and perceived work ability [24]. These distinct but intersecting influences are often overlooked in occupational health research, highlighting the need for a gender-sensitive and sex-aware approach.

MSDs further complicate this picture, as they are among the most prevalent occupational health issues in Europe and have been consistently linked to reduced work ability and quality of life [25-28]. Given their potential association with both PA levels and occupational demands, they represent a relevant outcome for understanding the interplay between LTPA, OPA, and workforce health. University staff represent a highly relevant study population given their exposure to elevated cognitive and sedentary workloads as well as their strategic role within knowledge-based economies. The university setting also provides a distinct methodological advantage, encompassing diverse occupational profiles with markedly different levels of OPA. PDI (Personal Docente e Investigador): Teaching and Research Staff, PI (Personal Investigador): Research Staff (not Principal Investigator), PTGAS (Personal Técnico, de Gestión, de Administración y de Servicios): Technical, Management, Administrative, and Services Staff often perform duties involving higher levels of physical effort. This internal heterogeneity allows for the examination of different domains of PA and work ability within a single institutional context, making it particularly well-suited for exploring the PA paradox.

Although physical fitness and higher LTPA levels have been associated with greater work ability and fewer musculoskeletal concerns [14,29,31], much of the existing literature does not adequately distinguish between OPA and LTPA or focuses predominantly on male or younger populations [13,32]. Furthermore, limited attention has been given to the role of sex and gender in moderating these relationships as well as the broader influence of social determinants of health, such as sex, gender, and occupational context, on PA behaviors and work-related outcomes [33-40]. Therefore, further research is needed to examine the interplay between LTPA, OPA, and work ability, incorporating a gender perspective and accounting for the influence of the social determinants of health. Such knowledge is critical to informing the development of healthy, equitable, and sustainable work environments.

### Objectives

On the basis of the aforementioned background, the primary objective of this study is to analyze the relationship between LTPA and work ability among university staff in the CAM, considering the potential modifying effect of OPA, and exploring differences by sex and gender. The secondary objectives are to (1) determine the prevalence of MSDs in this population and analyze their association with LTPA and OPA levels using validated questionnaires; (2) evaluate the relationship between both domains of PA and perceived physical and mental workload, adjusting for occupational role and sociodemographic factors; (3) examine the association between LTPA and OPA and self-perceived health-related quality of life (HRQoL); and (4) explore whether these associations vary according to sex, gender, and occupational category.

## Methods

### Study Design

This is a descriptive, cross-sectional, population-based study with a gender perspective. The study was registered on ClinicalTrials.gov before data collection (NCT06723808).

### Study Population and Setting

The study population consists of university staff working in the CAM. A representative sample of 885 workers from the University of Alcalá (UAH), Alcalá de Henares, Madrid, Spain, was selected. Participants include members of the PTGAS, PDI, and PI.

Inclusion criteria are (1) aged ≥18 years and (2) having sufficient proficiency in Spanish to complete study procedures. The questionnaire will be administered exclusively in Spanish, as it is the official and working language in the study setting. Consequently, only participants with adequate Spanish comprehension will be eligible. While this approach ensures the reliability of self-reported data, it may limit the generalizability of the findings to non–Spanish-speaking university staff.

Exclusion criteria include (1) cognitive impairment preventing comprehension of study information or completion of the survey, (2) severe neurological deterioration, and (3) diagnosed severe mental illness preventing the provision of informed consent.

### Recruitment and Sampling

Due to the institutional data protection constraints, it is not possible to distribute the survey invitation directly to all university employees. Consequently, a multichannel recruitment strategy is being implemented. The study information sheet and participation link are being disseminated via email to deans, faculties, departments, and the human resources department of the UAH, who may forward it to relevant bodies at their discretion. In addition, posters containing QR codes and brief study details are placed on bulletin boards across UAH buildings to increase visibility. Participants are also encouraged to share the study with colleagues to broaden its reach and improve participation rates, thereby introducing a secondary snowball component.

While this strategy incorporates elements of exponential snowball sampling, it is designed to promote broad participation across different occupational roles while complying with data protection policies. This approach facilitates access to diverse occupational profiles and enhances heterogeneity in the study sample.

### Sample Size

The sample size was calculated for a finite population using Microsoft Excel based on the total UAH workforce (N=3117). A minimum of 796 (25.53%) participants were required to achieve a 95% CI, 3% margin of error, and population proportion of 0.5. This last value was chosen due to the absence of previous prevalence data on the variables of interest, as it maximizes sample size under conditions of uncertainty and ensures sufficient statistical power across all outcomes. Accounting for a potential 10% dropout rate, the final estimated sample size was determined to be 885 (28.39%) participants.

### Study Measures

Data collection includes sociodemographic variables and validated questionnaires in Spanish to assess the primary and secondary study outcomes.

#### Primary Variables

The primary variables include LTPA, OPA, and work ability. LTPA and OPA are assessed using the Spanish version of the Global Physical Activity Questionnaire [41]. This tool assesses PA in 3 domains: occupational, active transport, and leisure, yielding weekly metabolic equivalent of task (MET) minutes for each. MET minutes are calculated by multiplying activity duration by the standard MET values.

The official Spanish version of the questionnaire, endorsed by the WHO, has shown good test-retest reliability (intraclass correlation coefficient=0.70-0.90) and moderate concurrent validity (Spearman ρ≈0.42) compared with the accelerometry in European and Latin American Spanish-speaking populations. Although it has not been formally validated in Spain, its widespread international use and standardization support its inclusion in this study [42].

Work ability is being assessed with the Spanish version of the Work Ability Index (WAI) [43]. The WAI measures 7 dimensions: current work ability compared to lifetime best, work ability in relation to job demands, physician-diagnosed illnesses, estimated work impairment due to illness, sick leave over the past year, prognosis of work ability over the next 2 years, and mental resources. Total scores classify work ability into 4 categories: poor (7-27), moderate (28-36), good (37-43), and excellent (43,45-49). The WAI has been adapted and endorsed by the Spanish National Institute for Safety and Health at Work (NTP 1147) for the Spanish occupational context [43]. It was validated in a sample of 1184 Spanish health center workers, demonstrating satisfactory reliability (intraclass correlation coefficient>0.75), a stable 2-factor structure, and convergent validity with measures of health and productivity, reflecting strong psychometric properties in occupational settings [44].

#### Secondary Variables

Secondary variables include the prevalence of MSDs, HRQoL, and physical and mental workload. MSDs are being evaluated using the Spanish version of the Standardized Nordic Questionnaire [45], which identifies symptoms across various anatomical regions and their functional impact. It includes symptom checklists and related consequences, such as reduced activity or health care visits. This questionnaire has been validated across multiple Spanish occupational samples. In a study of 526 nursing assistants in Asturias, the questionnaire demonstrated good test-retest reliability (Cohen κ=0.60-0.81) and internal consistency (Kuder-Richardson 20=0.74-0.87) [46]. In addition, in a separate validation among 312 professional musicians in Madrid, similar values were observed (Cohen κ=0.60-0.81; Kuder-Richardson 20=0.737-0.873), and construct validity was supported by significantly higher disability and pain scores in anatomical regions with reported symptoms [45]. The tool also demonstrated high feasibility, with an average completion time of 6 (SD 2) minutes [45].

HRQoL is being assessed using the Short Form-12 Health Survey [47], which covers 8 health dimensions across physical and mental domains. Scores are standardized to a population mean of 50 (SD 10); scores greater than 50 indicate better-than-average perceived health. The Short Form-12 Health Survey, validated in a representative Spanish adult population (n≈11,000), reported high internal consistency (Cronbach α=0.87 for the physical component summary and Cronbach α=0.86 for the mental component summary). It accounted for more than 90% of the variance observed in the Short Form-36 Health Survey, further supporting its reliability and construct validity for assessing HRQoL [48].

Workload is being evaluated using the National Aeronautics and Space Administration Task Load Index [49], which includes 6 subscales: mental, physical, and temporal demand, performance, effort, and frustration. After weighting each subscale, participants rate them on a scale of 0 to 100. Weighted scores are averaged to compute a total workload score. This index has been validated in Spain in a sample of 398 workers across 7 occupational sectors. The instrument demonstrated strong internal consistency (Cronbach α>0.80) and confirmed its intended factorial structure, supporting its suitability for assessing perceived workload in diverse occupational groups [50]. The Instituto Nacional de Seguridad y Salud en el Trabajo recommends its use to estimate perceived mental workload in real work environments due to its sensitivity to variation in cognitive and physical demands and its nonintrusive nature (NTP 544) [49].

Sociodemographic data collected include age, sex, gender, menopause status and related symptoms (if applicable), anthropometric variables, education level, work role (PTGAS, PDI, and PI), work schedule (split, continuous, or mixed), workday type (full time or part time), gross annual income, and number of cohabitants.

### Data Collection

Data collection began after receiving ethics approval in November 2024 (clinical research ethics committee: CEID/2024/3/061). At the time of manuscript submission, no statistical analyses had been conducted; this protocol describes the planned methodology in detail in accordance with the journal’s protocol submission requirements. Participants are completing an ad hoc online form created in Microsoft Forms, accessible on any internet-enabled device. The form includes all questionnaires and measures described earlier. Completion time is approximately 25 minutes. To optimize response rates and minimize perceived burden, this estimated completion time is clearly communicated in the invitation message. This duration was deemed acceptable for participants to balance respondent convenience with the need for comprehensive data collection. To further support participation, reminder messages will be sent at regular intervals to prompt responses and ensure questionnaire completion.

Data are stored automatically in a secure Microsoft Excel database, accessible only to the principal investigator, who ensures data completeness and accuracy, oversees coding, and securely maintains the dataset for subsequent statistical analysis.

### Planned Data Analysis

Descriptive analyses will summarize all variables, reporting means, medians, SDs, and quartiles for continuous variables and frequency distributions for categorical variables. Normality will be assessed using the Kolmogorov-Smirnov test.

To examine associations between PA levels (both OPA and LTPA) and the primary outcomes (work ability, musculoskeletal health, and quality of life), multiple linear and logistic regression model analyses will be performed, depending on the nature of the outcome variable. These models will adjust for relevant covariates, including age, sex, occupational group, and key social determinants of health. Moderation by gender will be assessed through the inclusion of interaction terms, and stratified analyses by sex and gender will be conducted to explore potential differential effects.

To account for the expected heterogeneity across occupational roles, key analyses will be stratified by job category (PDI or PI vs PTGAS). Occupational role will also be included as a covariate in multivariable regression models to adjust for its potential confounding effect. In addition, subgroup analyses will be conducted to explore interaction effects between occupational role and levels of OPA and LTPA on primary and secondary outcomes, including work ability, MSDs, perceived workload, and HRQoL. PA levels will be categorized using WHO-recommended cutoffs, with high PA defined as 600 MET-minutes per week or more, allowing for group comparisons (eg, high vs low OPA or LTPA) in relation to health and occupational outcomes.

Furthermore, mediation analyses will be conducted to evaluate whether MSDs mediate the relationship between PA and work ability. These analyses will follow sequential multiple regression models, applying the approach described by Preacher [51] and Hayes [52] and adapted for cross-sectional data. Model fit will be assessed using the *R*² coefficient and *F* test. Statistical significance will be set at *P*<.05, and 95% CIs will be reported for all regression coefficients.

This analytical approach aligns with a gender-sensitive framework, enabling the detection of differential effects and the potential moderating roles of gender in the relationships between PA, occupational factors, and health outcomes.

The online questionnaire is programmed so that all questions must be completed before submission, thereby minimizing the likelihood of missing data. Consequently, the proportion of missing values is expected to be negligible. In the unlikely event that incomplete data are detected (eg, due to technical errors or partial dropouts), a descriptive analysis of missingness will be performed. In such cases, listwise deletion will be applied.

All analyses will be conducted using SPSS Statistics (version 27.0; IBM Corp) by a blinded statistician working with coded data.

### Ethical Considerations

This study is being conducted in full accordance with the ethical principles of the Declaration of Helsinki. Participant confidentiality is ensured in compliance with Organic Law 3/2018 of December 5 on the Protection of Personal Data and Guarantee of Digital Rights, as well as Regulation (European Union) 2016/679 of the European Parliament and Council of April 27, 2016 (General Data Protection Regulation). All participants received an information sheet and provided voluntary, written informed consent prior to any data collection. No compensation was provided to participants for their participation in this study. The results will be reported in accordance with the STROBE (Strengthening the Reporting of Observational Studies in Epidemiology) guidelines [53].

## Results

This study was approved by the clinical research ethics committee of the UAH in November 2024 (CEID/2024/3/061), in compliance with the European data protection regulations. Recruitment began in December 2024 and is currently ongoing. As of July 2025, data collection was actively underway and is expected to continue until June 2027, the planned study completion date. Participants are completing the online questionnaire using Microsoft Forms, as detailed in the Methods section.

All responses are automatically collected and securely stored in a cloud-based spreadsheet accessible only to the principal investigator. Preliminary checks are being performed to ensure data completeness and consistency. No statistical analyses have been conducted to date, as data collection is still ongoing.

Upon completion of data collection, the final dataset will be analyzed following the predefined statistical plan. Study results, irrespective of their significance, will be disseminated in peer-reviewed national and international journals and presented at academic conferences. The dissemination strategy will adhere to the principles of a gender-sensitive and transparent reporting approach, aligned with current research integrity and open science standards.

## Discussion

### Anticipated Findings

The primary aim of this study is to investigate the relationship between LTPA and work ability, considering variations in OPA among university staff in the CAM, as well as to assess sex and gender differences within this population. In addition, this study explores potential sex and gender differences in these associations as well as the role of MSDs, physical and mental workload, and HRQoL.

Previous studies have identified positive associations between LTPA and work ability [31,32]. However, the so-called PA paradox suggests that high levels of OPA may attenuate or even negate the benefits of LTPA on work ability [54]. Holtermann et al [11] proposed several mechanisms to explain this paradox: OPA often lacks sufficient intensity or involves prolonged duration to improve cardiorespiratory fitness; it elevates heart rate and blood pressure during and after activity (over 24 hours); it is typically performed without adequate recovery; and it often occurs under conditions of low worker autonomy and high inflammatory load. Therefore, OPA may not only fail to confer the health benefits of LTPA but could, in some cases, be harmful.

In this context, research examining the influence of occupational demands on LTPA’s impact on work ability remains limited. Many existing studies have focused on male-dominated samples [32] or occupations characterized by high OPA [55]. In a recent study of the Korean workforce, Ko et al [54] observed that, while LTPA generally correlated positively with work ability among older adults in physically demanding jobs (high OPA), LTPA was paradoxically associated with lower productivity and work ability. Given the scarcity of similar studies in Spain, this study, which involves a heterogeneous sample of university staff with varied occupational demands, may help elucidate these associations in the local context.

Sex and gender are addressed in this study as central determinants of health, given their potential to shape PA behaviors, work ability, and health outcomes in complex and interrelated ways. Globally, women engage in less PA than men [2], a pattern also observed in Spain where inactivity rates range from 22.9% to 40.3% in men and 30.5% to 32.3% in women [2,56,57] and in the CAM region where 46.89% of the women report being sedentary compared to 30.61% of the men [3]. This disparity may reflect traditional caregiving roles, which often limit women’s opportunities for recreation (including LTPA) and self-care [22,23,30] as well as unique physiological and pathophysiological processes related to women’s health [24]. These factors suggest that women may derive fewer benefits from LTPA for work ability under equivalent occupational demands. Furthermore, life stages, such as menopause, are associated with decreased work ability [58,59], highlighting the relevance of exploring these associations in this subgroup.

MSDs, included in this study, are the most common and disabling occupational illnesses [27,60]. They are linked to reduced HRQoL [26], diminished functional capacity [61], and lower work ability [25-27,62]. While high OPA levels may contribute to MSD development [10], appropriate levels of LTPA could serve a protective role [1,63]. However, MSDs may also limit participation in LTPA, potentially creating a feedback loop that further compromises work ability. Similarly, high physical and mental workload is associated with lower LTPA participation [19], higher occupational demands, reduced work ability [64], and increased MSD prevalence, as reported by Saremi et al [65].

Finally, despite numerous workplace PA interventions aimed at improving work ability, many have failed to achieve outcomes beyond physical fitness [4]. This may be partly due to insufficient consideration of occupational demands and individual factors when designing LTPA programs [4]. The findings of this study will provide critical insights into the balance between LTPA and OPA, supporting the design of future interventions with a gender-sensitive approach. Ultimately, this could serve as a valuable tool to prevent noncommunicable diseases and promote optimal work ability among the working population in CAM.

### Strengths and Limitations

This protocol presents several strengths. First, it uses validated and widely used instruments to assess LTPA, OPA, work ability, and relevant covariates, alongside comprehensive sociodemographic data. The online, self-administered format of the questionnaire reduces potential bias associated with interviewer-led responses and facilitates participation across diverse occupational groups. By collecting key covariates, this study is designed to account for potential confounding factors, ensuring that observed associations are more likely attributable to the primary research objectives. In addition, the estimated sample size provides adequate statistical power to detect significant associations, and the inclusion of participants with varied occupations and occupational demands enhances the generalizability of findings to the broader CAM working population.

The primary limitation of this study is its cross-sectional design, which precludes causal inference between the variables of interest. Longitudinal and interventional studies will be necessary to establish causal relationships and evaluate the effectiveness of tailored PA interventions. In addition, the use of a nonprobability sampling strategy, necessitated by institutional data protection constraints, may introduce selection bias and limit representativeness. However, the multichannel recruitment approach has been carefully designed to promote broad dissemination across occupational groups, and the anticipated heterogeneity of the sample is considered sufficient for the study’s exploratory objectives. Moreover, the use of self-reported measures introduces the possibility of reporting bias, which could affect the accuracy of certain variables. Nevertheless, the use of validated instruments mitigates this concern to some extent. Finally, the absence of objective PA measurements (eg, accelerometers or step counters) may limit the precision of exposure assessment; however, this was a deliberate decision made during the study design phase to prioritize anonymity and feasibility, and it will be addressed in future phases involving subsamples with identifiable data.

As all questionnaire items are mandatory, missing data are not expected. Nonetheless, any unexpected data loss (eg, due to technical issues) will be managed using listwise deletion.

### Conclusions

This study aims to analyze the relationship between occupational and LTPA and work ability among university staff with diverse occupational demands in the CAM. By incorporating a gender-sensitive perspective, the findings are expected to provide insights into how different domains of PA, particularly leisure time versus occupational, interact with musculoskeletal health, mental and physical workload, and HRQoL.

The methodological design, grounded in validated measurement tools and a representative university population sample, is intended to generate robust and transferable evidence. By including both male and female participants, this study also seeks to address critical gaps in the literature regarding sex- and gender-based differences in the effects of PA and occupational demands.

The results of this research may inform the development of workplace health strategies that promote balanced PA, support sustainable work ability, and prevent noncommunicable diseases in knowledge-based work environments. Moreover, this study has the potential to guide future interventions and public policies aimed at reducing gender-based disparities in occupational health and equitable access to PA, in alignment with global health priorities and the Sustainable Development Goals.

## Abbreviations

CAM: Community of Madrid
HRQoL: health-related quality of life
LTPA: leisure-time physical activity
MET: metabolic equivalent of task
MSD: musculoskeletal disorder
OPA: occupational physical activity
PA: physical activity
PDI: teaching and research staff
PI: research staff
PTGAS: technical, management, administrative, and services staff
STROBE: Strengthening the Reporting of Observational Studies in Epidemiology
UAH: University of Alcalá
WAI: Work Ability Index
WHO: World Health Organization

## References

[1] Dhuli K, Naureen Z, Medori MC, Fioretti F, Caruso P, Perrone MA, Nodari S, Manganotti P, Xhufi S, Bushati M, Bozo D, Connelly ST (2022). Physical activity for health. J Prev Med Hyg.

[2] Guthold R, Stevens GA, Riley LM, Bull FC (2020). Global trends in insufficient physical activity among adolescents: a pooled analysis of 298 population-based surveys with 1·6 million participants. Lancet Child Adolesc Health.

[3] (2020). Sedentarismo según sexo y comunidad autónoma. Población de 15 y más años. Instituto Nacional de Estadística.

[4] Mänttäri S, Oksa J, Lusa S, Korkiakangas E, Punakallio A, Oksanen T, Laitinen J (2021). Interventions to promote work ability by increasing physical activity among workers with physically strenuous jobs: a scoping review. Scand J Public Health.

[5] (2017). World population prospects: the 2017 revision. United Nations.

[6] Recomendaciones Mundiales sobre Actividad Física para la Salud. Organización Mundial de la Salud.

[7] (2008). 2008 physical activity guidelines for Americans. US Department of Health and Human Services.

[8] Yu X, Hao L, Crainiceanu C, Leroux A (2022). Occupational determinants of physical activity at work: evidence from wearable accelerometer in 2005-2006 NHANES. SSM Popul Health.

[9] Moreira S, Criado MB, Santos PC, Ferreira MS, Gonçalves C, Machado J (2022). Occupational health: physical activity, musculoskeletal symptoms and quality of life in computer workers: a narrative review. Healthcare (Basel).

[10] Nordander C, Hansson GÅ, Ohlsson K, Arvidsson I, Balogh I, Strömberg U, Rittner R, Skerfving S (2016). Exposure-response relationships for work-related neck and shoulder musculoskeletal disorders--analyses of pooled uniform data sets. Appl Ergon.

[11] Holtermann A, Krause N, van der Beek AJ, Straker L (2018). The physical activity paradox: six reasons why occupational physical activity (OPA) does not confer the cardiovascular health benefits that leisure time physical activity does. Br J Sports Med.

[12] Nawrocka A, Garbaciak W, Cholewa J, Mynarski W (2018). The relationship between meeting of recommendations on physical activity for health and perceived work ability among white-collar workers. Eur J Sport Sci.

[13] Ribas TM, Teodori RM, Mescolotto FF, Montebelo MI, Baruki SB, Pazzianotto-Forti E (2021). Impact of physical activity levels on musculoskeletal symptoms and absenteeism of workers of a metallurgical company. Rev Bras Med Trab.

[14] López-Bueno R, Smith L, Andersen LL, López-Sánchez GF, Casajús JA (2020). Association between physical activity and sickness absenteeism in university workers. Occup Med (Lond).

[15] Dallmeyer S, Wicker P, Breuer C (2023). The relationship between leisure-time physical activity and job satisfaction: a dynamic panel data approach. J Occup Health.

[16] Bailey R, Hillman C, Arent S, Petitpas A (2013). Physical activity: an underestimated investment in human capital?. J Phys Act Health.

[17] van den Berg TI, Elders LA, de Zwart BC, Burdorf A (2009). The effects of work-related and individual factors on the Work Ability Index: a systematic review. Occup Environ Med.

[18] Andersen LL, Fallentin N, Thorsen SV, Holtermann A (2016). Physical workload and risk of long-term sickness absence in the general working population and among blue-collar workers: prospective cohort study with register follow-up. Occup Environ Med.

[19] de Vries JD, Bakker AB (2022). The physical activity paradox: a longitudinal study of the implications for burnout. Int Arch Occup Environ Health.

[20] Bull FC, Al-Ansari SS, Biddle S, Borodulin K, Buman MP, Cardon G, Carty C, Chaput JP, Chastin S, Chou R, Dempsey PC, DiPietro L, Ekelund U, Firth J, Friedenreich CM, Garcia L, Gichu M, Jago R, Katzmarzyk PT, Lambert E, Leitzmann M, Milton K, Ortega FB, Ranasinghe C, Stamatakis E, Tiedemann A, Troiano RP, van der Ploeg HP, Wari V, Willumsen JF (2020). World Health Organization 2020 guidelines on physical activity and sedentary behaviour. Br J Sports Med.

[21] Katzmarzyk PT, Powell KE, Jakicic JM, Troiano RP, Piercy K, Tennant B, 2018 Physical Activity Guidelines Advisory Committee* (2019). Sedentary behavior and health: update from the 2018 physical activity guidelines advisory committee. Med Sci Sports Exerc.

[22] Fredman L, Bertrand RM, Martire LM, Hochberg M, Harris EL (2006). Leisure-time exercise and overall physical activity in older women caregivers and non-caregivers from the Caregiver-SOF Study. Prev Med.

[23] Sanjuán-Quiles Á, Alcañiz-Garrán MD, Montejano-Lozoya R, Ramos-Pichardo LD, García-Sanjuán S (2023). La perspectiva de las personas cuidadoras desde un análisis de género. Rev Esp Salud Publica.

[24] Gjellestad M, Haraldstad K, Enehaug H, Helmersen M (2023). Women's health and working life: a scoping review. Int J Environ Res Public Health.

[25] Rosado AS, Baptista JS, Guilherme MN, Guedes JC, Arezes PM, Baptista JS, Melo RB, Branco JC, Carneiro P, Colim A, Costa N (2023). Economic impact of work-related musculoskeletal disorders—a systematic review. Occupational and Environmental Safety and Health IV.

[26] Chang YF, Yeh CM, Huang SL, Ho CC, Li RH, Wang WH, Tang FC (2020). Work ability and quality of life in patients with work-related musculoskeletal disorders. Int J Environ Res Public Health.

[27] Phongamwong C, Deema H (2015). The impact of multi-site musculoskeletal pain on work ability among health care providers. J Occup Med Toxicol.

[28] de Pereira MA, Carnide F, Cotrim T, Arezes PM, Melo RB, Carneiro P, Branco JC (2024). Work ability determinants in industry: what are the gaps? A narrative review. Occupational and Environmental Safety and Health V.

[29] Søgaard K, Sjøgaard G (2017). Physical activity as cause and cure of muscular pain: evidence of underlying mechanisms. Exerc Sport Sci Rev.

[30] Fredman L, Cauley JA, Hochberg M, Ensrud KE, Doros G, Study of Osteoporotic Fractures (2010). Mortality associated with caregiving, general stress, and caregiving-related stress in elderly women: results of caregiver-study of osteoporotic fractures. J Am Geriatr Soc.

[31] Norheim KL, Samani A, Hjort Bønløkke J, Omland Ø, Madeleine P (2019). Physical-work ability and chronic musculoskeletal complaints are related to leisure-time physical activity: cross-sectional study among manual workers aged 50-70 years. Scand J Public Health.

[32] Päivärinne V, Kautiainen H, Heinonen A, Kiviranta I (2019). Relationships of leisure-time physical activity and work ability between different occupational physical demands in adult working men. Int Arch Occup Environ Health.

[33] Gidlow C, Johnston LH, Crone D, Ellis N, James D (2006). A systematic review of the relationship between socio-economic position and physical activity. Health Educ J.

[34] (2007). Subsanar las desigualdades en una generación : alcanzar la equidad sanitaria actuando sobre los determinantes sociales de la salud : resumen analítico del informe final. Organización Mundial de la Salud.

[35] Ruiz Álvarez M, Aginagalde Llorente AH, Del Llano Señarís JE (2022). Los determinantes sociales de la salud en España (2010-2021): una revisión exploratoria de la literatura. Rev Esp Salud Publica.

[36] O'Donoghue G, Kennedy A, Puggina A, Aleksovska K, Buck C, Burns C, Cardon G, Carlin A, Ciarapica D, Colotto M, Condello G, Coppinger T, Cortis C, D'Haese S, De Craemer M, Di Blasio A, Hansen S, Iacoviello L, Issartel J, Izzicupo P, Jaeschke L, Kanning M, Ling F, Luzak A, Napolitano G, Nazare JA, Perchoux C, Pesce C, Pischon T, Polito A, Sannella A, Schulz H, Simon C, Sohun R, Steinbrecher A, Schlicht W, MacDonncha C, Capranica L, Boccia S (2018). Socio-economic determinants of physical activity across the life course: a "DEterminants of DIet and Physical ACtivity" (DEDIPAC) umbrella literature review. PLoS One.

[37] D'Amore C, Saunders S, Bhatnagar N, Griffith L, Richardson J, Beauchamp M (2023). Determinants of physical activity in community-dwelling older adults: an umbrella review. Int J Behav Nutr Phys Act.

[38] Moreno-Llamas A, García-Mayor J, De la Cruz-Sánchez E (2022). Gender inequality is associated with gender differences and women participation in physical activity. J Public Health (Oxf).

[39] Garg S (2023). Gender differences in pathways influencing leisure time physical activity: a structural equation analysis. Diabetes Metab Syndr.

[40] Rosselli M, Ermini E, Tosi B, Boddi M, Stefani L, Toncelli L, Modesti PA (2020). Gender differences in barriers to physical activity among adolescents. Nutr Metab Cardiovasc Dis.

[41] Cuestionario Mundial sobre Actividad Física (GPAQ). Organización Mundial de la Salud.

[42] Keating XD, Zhou K, Liu X, Hodges M, Liu J, Guan J, Phelps A, Castro-Piñero J (2019). Reliability and concurrent validity of global physical activity questionnaire (GPAQ): a systematic review. Int J Environ Res Public Health.

[43] (2020). NTP 1147: Work Ability Index – versión española. Instituto Nacional de Seguridad y Salud en el Trabajo.

[44] Mateo Rodríguez I, Knox EC, Oliver Hernández C, Daponte Codina A, The esTAR Group (2021). Psychometric properties of the work ability index in health centre workers in Spain. Int J Environ Res Public Health.

[45] Gómez-Rodríguez R, Díaz-Pulido B, Gutiérrez-Ortega C, Sánchez-Sánchez B, Torres-Lacomba M (2020). Cultural adaptation and psychometric validation of the standardised Nordic questionnaire Spanish version in musicians. Int J Environ Res Public Health.

[46] Mateos-González L, Rodríguez-Suárez J, Llosa JA, Agulló-Tomás E (2024). Spanish version of the Nordic Musculoskeletal Questionnaire: cross-cultural adaptation and validation in nursing aides. An Sist Sanit Navar.

[47] Schmidt S, Vilagut G, Garin O, Cunillera O, Tresserras R, Brugulat P, Mompart A, Medina A, Ferrer M, Alonso J (2012). Normas de referencia para el Cuestionario de Salud SF-12 versión 2 basadas en población general de Cataluña. Med Clin (Barc).

[48] Vilagut G, Valderas J, Ferrer M, Garin O, López-García E, Alonso J (2008). [Interpretation of SF-36 and SF-12 questionnaires in Spain: physical and mental components]. Med Clin (Barc).

[49] NTP 544: Estimación de la carga mental de trabajo: el método NASA-TLX - Año 2000. Instituto Nacional de Seguridad y Salud en el Trabajo.

[50] Eva DR, Susana RV, Jesus MG, Lourdes LM (2010). Revista de Psicología del Trabajo y de las Organizaciones. Rev Psicol Trab Organ.

[51] Preacher KJ, Hayes AF (2008). Asymptotic and resampling strategies for assessing and comparing indirect effects in multiple mediator models. Behav Res Methods.

[52] Hayes AF (2018). Introduction to Mediation, Moderation, and Conditional Process Analysis, First Edition: A Regression-Based Approach. 2nd edition.

[53] von Elm E, Altman DG, Egger M, Pocock SJ, Gøtzsche PC, Vandenbroucke JP (2007). Strengthening the reporting of observational studies in epidemiology (STROBE) statement: guidelines for reporting observational studies. BMJ.

[54] Ko H, Kim D, Cho SS, Kang MY (2023). The physical activity paradox in relation to work ability and health-related productivity loss in Korea. Epidemiol Health.

[55] Calatayud J, Jakobsen MD, Sundstrup E, Casaña J, Andersen L (2015). Dose-response association between leisure time physical activity and work ability: cross-sectional study among 3000 workers. Scand J Public Health.

[56] Guthold R, Stevens GA, Riley LM, Bull FC (2018). Worldwide trends in insufficient physical activity from 2001 to 2016: a pooled analysis of 358 population-based surveys with 1·9 million participants. Lancet Glob Health.

[57] Determinantes de salud (sobrepeso, consumo de fruta y verdura, tipo de lactancia, actividad física). Instituto Nacional de Estadística.

[58] Geukes M, van Aalst MP, Robroek SJ, Laven JS, Oosterhof H (2016). The impact of menopause on work ability in women with severe menopausal symptoms. Maturitas.

[59] Moilanen J, Aalto AM, Hemminki E, Aro A, Raitanen J, Luoto R (2010). Prevalence of menopause symptoms and their association with lifestyle among Finnish middle-aged women. Maturitas.

[60] Bayattork M, Jakobsen MD, Sundstrup E, Seidi F, Bay H, Andersen LL (2019). Musculoskeletal pain in multiple body sites and work ability in the general working population: cross-sectional study among 10,000 wage earners. Scand J Pain.

[61] Lakke SE, Wittink H, Geertzen JH, van der Schans CP, Reneman M (2012). Factors that affect functional capacity in patients with musculoskeletal pain: a Delphi study among scientists, clinicians, and patients. Arch Phys Med Rehabil.

[62] Ilmarinen JE (2001). Aging workers. Occup Environ Med.

[63] Lin I, Wiles L, Waller R, Goucke R, Nagree Y, Gibberd M, Straker L, Maher CG, O'Sullivan PP (2020). What does best practice care for musculoskeletal pain look like? Eleven consistent recommendations from high-quality clinical practice guidelines: systematic review. Br J Sports Med.

[64] Ghanavati FK, Choobineh A, Keshavarzi S, Nasihatkon A, Jafari Roodbandi AS (2019). Assessment of mental workload and its association with work ability in control room operators. Med Lav.

[65] Saremi M, Madvari RF, Khoshakhlagh A, Laal F (2021). The Relationship between Work Ability Index (WAI), Mental Workload and Musculoskeletal Disorders (MSDs) of Firefighters in Tehran, Iran. Iran J Public Health.

